# Expression patterns of candidate genes for the *Lr46/Yr29* “slow rust” locus in common wheat (*Triticum aestivum* L.) and associated miRNAs inform of the gene conferring the *Puccinia triticina* resistance trait

**DOI:** 10.1371/journal.pone.0309944

**Published:** 2024-09-06

**Authors:** Julia Spychała, Agnieszka Tomkowiak, Aleksandra Noweiska, Roksana Bobrowska, Sandra Rychel-Bielska, Jan Bocianowski, Łukasz Wolko, Przemysław Łukasz Kowalczewski, Marcin Nowicki, Michał Tomasz Kwiatek

**Affiliations:** 1 Department of Genetics and Plant Breeding, Poznań University of Life Sciences, Poznań, Poland; 2 Plant Breeding and Acclimatization Institute - National Research Institute in Radzików, Poznań Division, Department of Oilseed Crops, Poznań, Poland; 3 Department of Genetics, Plant Breeding and Seed Production, Wrocław University of Environmental and Life Sciences, Wrocław, Poland; 4 Department of Mathematical and Statistical Methods, Poznań University of Life Sciences, Poznań, Poland; 5 Department of Biochemistry and Biotechnology, Poznań University of Life Sciences, Poznań, Poland; 6 Department of Food Technology of Plant Origin, Poznań University of Life Sciences, Poznań, Poland; 7 Department of Entomology and Plant Pathology, Institute of Agriculture, University of Tennessee, Knoxville, Tennessee, United States of America; 8 Plant Breeding and Acclimatization Institute - National Research Institute in Radzików, Radzikow, Poland; Indian Agricultural Research Institute, INDIA

## Abstract

Leaf rust caused by *Puccinia triticina* (*Pt*) is one of the most impactful diseases causing substantial losses in common wheat (*Triticum aestivum* L.) crops. In adult plants resistant to *Pt*, a horizontal adult plant resistance (APR) is observed: APR protects the plant against multiple pathogen races and is distinguished by durable persistence under production conditions. The *Lr46/Yr29* locus was mapped to chromosome 1B of common wheat genome, but the identity of the underlying gene has not been demonstrated although several candidate genes have been proposed. This study aimed to analyze the expression of nine candidate genes located at the *Lr46/Yr29* locus and their four complementary miRNAs (tae-miR5384-3p, tae-miR9780, tae-miR9775, and tae-miR164), in response to *Pt* infection. The plant materials tested included five reference cultivars in which the molecular marker *csLV46G22* associated with the *Lr46/Yr29*-based *Pt* resistance was identified, as well as one susceptible control cultivar. Biotic stress was induced in adult plants by inoculation with fungal spores under controlled conditions. Plant material was sampled before and at 6, 12, 24, 48 hours post inoculation (hpi). Differences in expression of candidate genes at the *Lr46/Yr29* locus were analyzed by qRT-PCR and showed that the expression of the genes varied at the analyzed time points. The highest expression of *Lr46/Yr29* candidate genes (*Lr46-Glu1*, *Lr46-Glu2*, *Lr46-Glu3*, *Lr46-RLK1*, *Lr46-RLK2*, *Lr46-RLK3*, *Lr46-RLK4*, *Lr46-Snex*, and *Lr46-WRKY*) occurred at 12 and 24 hpi and such expression profiles were obtained only for one candidate gene among the nine genes analyzed (*Lr46-Glu2*), indicating that it may be a contributing factor in the resistance response to *Pt* infection.

## Introduction

Common wheat (*Triticum aestivum* L.) is one of staple crop species of enormous global importance. Worldwide, wheat cultivation is extremely relevant as it is connected with the crop’s large share in human and animal nutrition. Currently, the primary directions of wheat breeding focus mainly on high yield, high commercial quality, and resistance to biotic and abiotic stresses [1–3]. Among the most threatening diseases leading to substantial losses in world’s wheat crops is leaf rust caused by the fungus *Puccinia triticina* Eriks (*Pt*). It occurs more frequently than other rust diseases and is comparatively more prevalent worldwide [4]. Wheat leaf rust contributes substantially to the crop losses [4, 5]. *Pt* is an obligate parasite capable of producing infectious urediniospores that can spread over long distances, which may result in an epidemic of leaf rust on a national or even continental scale [6]. Scientists’ efforts are being focused on the possible future threats posed by climate change that renders negative impacts on global crop production and food security. Modern breeding technologies and biotechnology strategies are valuable tools to create climate-smart crops. In addition, to understand plant responses under various abiotic and biotic stresses, the most urgent need currently is to investigate their respective genetic basis and molecular mechanisms [7]. In 2020, Prasad et al. [5] analyzed hundreds of major scientific reports on mechanisms of plant resistance to fungal pathogens, including *Pt*. So far, the number of identified leaf rust resistance genes (*Lr*) is limited, and potential co-expression data have not yet been sufficiently examined. To understand the variability of *Lr* genes and the molecular mechanisms used to activate their expression after infection, the pursuit to clone more *Lr* genes is essential. The search for diverse and durable resistance to *Pt* must continue using advanced tools and techniques, including molecular methods [5].

Common wheat (*Triticum aestivum* L.) is an allohexaploid species (2n = 6× = 42 chromosomes; AABBDD) as a result of its dynamic domestication history [8]. This polyploidy is the result of two rounds of hybridization: the first interspecific between *T*. *urartu* (2n = 2× = 14 chromosomes; AA) and *Aegilops* spp. (genome BB), then between *T*. *turgidum* (2n = 4× = 28 chromosomes; AABB) and *A*. *tauschii* (2n = 2× = 14 chromosomes; DD) [9, 10]. The resulting common wheat genome is exceptionally large (approximately 16 Gbp) and, similar to other large plant genomes, its repeat sequences (including retrotransposons) account for as much as about 85%. Over the past decades, numerous studies have led to the identification of approximately 100 *Lr* genes and many important quantitative trait loci (QTLs) on various chromosomes of wheat genome [11, 12]. However, due to the size and complexity of the wheat genome, only a few *Lr* genes have been cloned so far. These include seven genes (*Lr1*, *Lr10*, *Lr21*, *Lr34*, *Lr42*, *Lr47*, and *Lr67*) cloned by classical map-based cloning and four genes obtained by rapid gene-cloning approaches (*Lr9*, *Lr13*, *Lr14a*, and *Lr22a*) [5, 13]. Genetically determined resistance to *Pt* has been characterized both in young plants (seedling resistance; SR) and plants at the mature stage (adult plant resistance; APR). APR is characterized by a comparatively greater persistence than SR [14]. Genes that underlie APR confer quantitative resistance, also known as horizontal resistance, that provides trait stability and reduced risk of future susceptibility. Majority of characterized *Lr* genes confer the SR type resistance at the seedling stage and are race-specific; exceptions include the few known APR genes such as *Lr34/Yr18/Sr57*, *Lr46/Yr29/Sr58*, *Lr67/Yr46/Sr55*, and *Lr68* [15–18]. In the APR-type *Pt*-resistant wheat cultivars, the observed slow rust impacts manifest as partial but durable *Pt* resistance [19]. An example of a gene with such resistance is the well-studied *Lr34* gene [20–25]. *Lr* genes have been successfully used in many breeding programs to develop novel wheat cultivars with improved pathogen resistance. Currently, only a few APR genes, such as *Lr34* and *Lr46*, are meaningfully useful in *Pt-*resistance breeding and are widely used in wheat breeding [25–27].

The *Lr46* gene was first described in the cultivar ’Pavon 76’ [28]. Wheat genotypes showing *Lr46/Yr29* are characterized by resistance to leaf and stripe rust pathogens; in addition, they have been characterized as donors of resistance to powdery mildew (*Pm39*) and stem rust pathogens (*Sr58*) [6]. The associated locus *Lr46/Yr29/Sr58/Pm39* was mapped to chromosome 1BL, but the specific genes are yet to be identified. Based on high-resolution mapping of wheat *QYr*.*ucw-1BL*, 13 candidate genes for *Lr46/Yr29* were selected and analyzed [29]. However, it remains unclear whether this locus of resistance to multiple pathogens is the result of the pleiotropic action of a single causal gene or a small cluster of tightly linked genes [30]. Detailed functional characterization of the *Lr46/Yr29* is essential to determine the allelic variability of the gene(s) that underlies the *Pt* resistance trait and to establish the molecular mechanism(s) of such a function [20].

Resistance and susceptibility are regulated by mechanisms for rapid recognition of invading pathogens and efficient activation of host plant defense mechanisms. Small non-coding RNAs, which can be subdivided into microRNAs (miRNAs) and small interfering RNAs (siRNAs), are global regulators of gene expression. In recent years, many miRNAs specific for plant development and various biotic and abiotic stress responses have been identified in wheat. In plants, regulation of expression by miRNAs is mainly through post-transcriptional regulation. miRNAs regulate not only the processes of plant growth and development but also actively participate in plant responses to biotic stress agents [31–34]. Plant miRNAs are small molecules of approximately 21 bp (base pairs) [35]. They recognize specific target mRNAs based on sequence complementarity and repress expression of the target gene(s) by targeting the mRNA for degradation or by inhibiting translation [36–38]. Obtaining a holistic knowledge of the functional dynamics and conservation of miRNAs in wheat can provide valuable information for the development of cultivars with improved stress resistance and yield [31, 39].

In this study, we analyzed the expression levels of nine candidate genes encompassed by *Lr46/Yr29*, selected based on the work by Cobo et al. [29]. These candidate genes mapped the *QYr*.*ucw-1BL* region to chromosome 1BL, which overlapped with the current genetic maps of the *Pt* resistance gene *Yr29* (also known as *Lr46*) [28]. The results suggest that QTL (*QYr*.*ucw-1BL*) and *Lr46/Yr29* map to the same gene conferring similar resistance responses. Previous data [29] highlighted the need to investigate the trait’s expression in different genotypes with and without pathogen inoculation to determine whether there are differences in the expression levels among candidate genes for *Lr46/Yr29*. As a result, 13 candidate genes were identified for *Lr46/Yr29*, but three of them were discarded (TraesCS1B02G454300, TraesCS1B02G454700, and TraesCS1B02G454900), because the first gene is annotated as a transposable element and there are no exome capture data; the other two genes were excluded because they are likely to be unexpressed pseudogenes. To confirm the obtained amplicons, we performed Sanger sequencing of all amplified products and compared the assembled sequences with homologues of genes located on other chromosomes: 1A, 1D, 2A, 2B, and 2D. Furthermore, we used stem-loop Reverse Transcription PCR (RT-PCR) and droplet digital PCR (ddPCR) methods to analyze the expression of miRNA molecules complementary to candidate genes for *Lr46/Yr29*, which can influence or modulate their expression over time.

## Materials and methods

### Plant growth, pathogen inoculation, and leaf sample collection

Plant materials consisted of six common wheat cultivars whose seeds were obtained from the National Small Grains Collection (Agricultural Research Station in Aberdeen, WA, USA). Five of the common wheat cultivars tested were found to be highly valuable sources of *Pt* resistance genes: spring cultivars Artigas (“PI 192535”), NP846 (“PI 322263”), Glenlea (“CItr 17272”), Lerma Rojo (“CItr 13651”), and one winter wheat cultivar TX89D6435 (“PI 584759”). The susceptibility control (lack of molecular markers linked to *Lr34*, *Lr46*, or *Lr67*) was the spring cultivar Artigas* (“PI 73046”) [20, 40]. Identification of molecular markers for the *Lr46/Yr29* locus of Artigas* and the listed *Pt*-resistant wheat cultivars analyzed in this paper was recently carried out by our research team [20]. The *csLV46G22* marker linked to the *Lr46/Yr29* locus (E. Lagudah, unpublished data) was identified in all five resistant wheat genotypes [20].

The experiment was conducted in a growth chamber under controlled conditions: the temperature was set at 18 °C during the day and 16 °C at night with a 16-hour photoperiod, a relative humidity of 60 to 70% was established. In addition, the emission spectrum of the light source was fixed with a photon flux of 572 μE. One month after setting up the experiment, the temperature in the phytotron was raised to 20 °C and 17 °C during the day and night, respectively. Plants were grown to the adult plant stage, i.e., the formation of the flag leaf; plants were inoculated at 34 days after sowing. Cultivars at the adult plant stage were inoculated with *Pt* by spraying a suspension of urediniospores at a concentration of approximately 5 × 10^5^ spores/mL. Inoculum suspension was an equimolar mixture of four *Pt* strains suspended in water with 1% v/v Tween 20 reagent and prepared immediately before inoculation [40, 41]. Fungal spores were collected from infected field experiments located in various parts of Poland [40]. Samples of leaf tissue fragments were collected at five time points: 0 (before inoculation), 6, 12, 24, and 48 hours post inoculation (hpi), in three biological replicates. Leaf tissue samples were immediately frozen in liquid nitrogen and stored at -80 °C.

### Candidate gene expression analysis using Quantitative Reverse Transcription PCR (qRT-PCR)

Based on the work of Cobo et al. [29], 10 candidate genes for the *Lr46/Yr29* gene (Table 1) whose sequences were found in the Ensembl Plants database for common wheat (https://plants.ensembl.org/Triticum_aestivum/Info/Index) were selected and downloaded in FASTA format. These sequences were used to design primers for qRT-PCR reactions, using the Primer3Plus tool (https://www.bioinformatics.nl/cgi-bin/primer3plus/primer3plus.cgi). The *Lr46/Yr29* region described by Cobo et al. [29] involves four copies of the *RECEPTOR-LIKE PROTEIN KINASE* (*RLK*) gene, three copies of *GLUCAN ENDO-1*,*3-BETA-GLUCOSIDASES* (*Glu*), as well as one copy each of *SORTING NEXIN* (*Snex*), *SUGAR TRANSPORTER* (*STr*), and a *TRANSCRIPTION FACTOR CARRYING THE WRKY DOMAIN* (*WRKY*). Of all the candidate genes listed, only the *STr* gene has no other close analogues in the reference wheat genome [42]. The sequences of the two candidate genes *Lr46-Snex* and *Lr46-WRKY* were similar to one another, which allowed universal primers to be designed to detect any transcript including homologues in other chromosomes. For the *Lr46-Snex* gene, a pair of universal primers was designed to allow detection of potential homologue transcripts on chromosomes 1A, 1B, and 1D. Similar was true for the *Lr46-WRKY* gene, for which a pair of universal primers was designed to allow the detection of potential gene transcripts of homologues on chromosomes 1B and 1D. Regarding the *RLK* and *Glu* genes, very similar gene arrangements are found on the other two chromosomes: 1A and 1D, in the region similar to 1B chromosome. Therefore, a comparison was made of only those few copies of genes found in the *QYr*.*ucw-1BL* region described, on chromosome 1B. These genes are so highly differentiated that it is not possible to design universal primers for them. Based on the *T*. *aestivum* reference genome sequence (taxid: 4565), primers were designed. Isolation of total RNA from leaf tissue samples collected as biological replicates and at variable time points (0, 6, 12, 24, and 48 hpi) was performed using the Maxwell RSC Plant RNA Kit isolation (Promega, Madison, WI, USA). The concentration and purity of isolated total RNA were assessed using a NanoDrop spectrophotometer and A_260_/A_280_ ratio. First-strand cDNA synthesis was performed using the iScript^™^ Reverse Transcription Supermix kit for RT-qPCR (Bio-Rad, Hercules, CA, USA), according to the protocol provided by the manufacturer. A temperature gradient PCR was performed for each investigated amplicon (each primer pair) to optimize the thermal profile applied. The gradient was set in the temperature range from 50 °C to 58 °C. The optimized amplification temperature was set at 53.5 °C after obtaining a specific product in a 2% agarose gel. In order to perform amplification standard curves, the amplicons obtained were individually purified using the QIAquick PCR Purification Kit (Qiagen Inc., Hilden, Germany) according to the protocol provided by the company. In the next step, a series of dilutions were performed for each purified amplicon to obtain standard curves of qRT-PCR efficiency (%E) and R^2^ values (Table 2). The standard curves were developed according to the protocol described previously [43].

**Table 1:** Presentation of 10 genes located in the candidate region for *Lr46/Yr29* gene and *QYr*.*ucw-1BL* region, predicted function and their localization on reference wheat genome TGACv2 (GCA_900241085.1).

No.	Gene ID	Gene abbreviation	Gene predicted function	Physical localization
**1.**	TraesCS1B02G453900.1	***Lr46-Glu1***	Glucan endo-1,3- β -glucosidase	1B: 669,922,599–669,924,501
**2.**	TraesCS1B02G454200.1	***Lr46-Glu2***	Glucan endo-1,3- β -glucosidase	1B: 670,142,374–670,144,629
**3.**	TraesCS1B02G454500.1	***Lr46-Glu3***	Glucan endo-1,3- β -glucosidase	1B: 670,158,858–670,161,009
**4.**	TraesCS1B02G454000.2	***Lr46-RLK1***	RLK (receptor-like kinase)	1B: 670,025,362–670,028,705
**5.**	TraesCS1B02G454100.1	***Lr46-RLK2***	RLK (receptor-like kinase)	1B: 670,034,245–670,037,207
**6.**	TraesCS1B02G454400.1	***Lr46-RLK3***	RLK (receptor-like kinase)	1B: 670,152,915–670,155,867
**7.**	TraesCS1B02G454600.1	***Lr46-RLK4***	RLK (receptor-like kinase)	1B: 670,164,777–670,167,783
**8.**	TraesCS1B02G454800.2	***Lr46-STr***	Sugar transporter	1B: 670,185,922–670,187,880
**9.**	TraesCS1B02G453700.1	***Lr46-Snex***	Sorting nexin	1B: 669,895,813–669,902,629
**10.**	TraesCS1B02G455000.1	***Lr46-WRKY***	WRKY transcription factor	1B: 670,202,181–670,203,493

**Table 2:** Name and sequences of designed primers based on candidate gene sequences for *Lr46/Yr29*. The table also shows qPCR efficiency (%E) and R^2^ values by performing standard curves.

Gene abbreviation	Primer sequence (5’- 3’)	Product size (bp)	Amplification efficiency (%E)	R^2^	T_m_ (°C)
***Lr46-Glu1***	F GGTGCAGAGCAATGTGAA R AGCTGAGAGTTTAGTTGG	102	84.4	0.999	84.5
***Lr46-Glu2***	F TATCTCTTGTTCCGCCCC R CCATCGCATAGTACACAG	103	84.3	0.998	84.5
***Lr46-Glu3***	F ACTCCAGACGTCATTCCC R GAACCGGTCTGTCGGAAAA	99	93.1	0.999	85.5
***Lr46-RLK1***	F ACGGGAAGGAAGAACAAT R ATCCATCATGTCCAACACC	105	109.4	0.998	79.5
***Lr46-RLK2***	F TGAGATCGTGACGGGAAG R CTAGCATCTCCAGTAGTGT	114	95.3	0.998	83.5
***Lr46-RLK3***	F CAGGGACCTTAAAGCTAAT R GGTTTGAGTATGAGTGTG	109	92.0	0.999	80.5
***Lr46-RLK4***	F TTCAGCTTTGGCGTATTGCAAC R ACGGGTCTAGCATCTCCAGT	101	91.3	0.998	83.0
***Lr46-STr***	F GGCCGTGAACGTGTATAT R GTGTCGGTGCCATTTCAG	107	-	-	-
***Lr46-Snex***	F CTTTGATAGTTCTGTTTCGC R TTTGTTTTGGCAAGTGGG	103	92.1	1.000	81.5
***Lr46-WRKY***	F TTTCTTCGCCTCTTTTGAC R GTGGAACCAATTCTCGTA	120	94.2	0.999	82.5

qRT-PCR was performed using iTaq Universal SYBR Green Supermix and CFX96 Touch Real-Time PCR Detection System (Bio-Rad, Hercules, CA, USA). A negative No Template Control (NTC) was also performed for each analyzed gene. The composition of the qRT-PCR reaction mix was as follows: supermix—5 μL, primers (10 μM)—0.5 μL each, nuclease-free water—3 μL, and cDNA template -1 μL. Following current MIQUE recommendations on the validity of real-time PCR analyses, three biological replicates were used for each gene type/timepoint for a given wheat cultivar, and three technical repeats were prepared for each [44]. The following temperature profile was used: initial denaturation for 3 min at 95 °C; then 40 cycles: denaturation for 10 sec at 95 °C, annealing of primers for 30 sec at 53.5 °C. Melt stage (melt curve): melting temperature range 65 °C to 90 °C; every 5 seconds the temperature was increased by 0.5 °C and a fluorescence measurement was taken. Data analyses were performed using Bio-Rad CFX Maestro software and the Gene Study tool (Bio-Rad Laboratories, Inc., Hercules, CA, USA), which allowed to compare the expression of all genes at different time points of each cultivar.

### Reference genes for Real-Time Quantitative Reverse Transcription PCR (qRT-PCR)

Based on literature data, four reference genes were selected for stable expression: *TUBβ*, *ARF*, *RLI* [45], and *EF2-1* [46]. After analyzing the standard curves for these reference genes, two genes, *TUBβ* and *ARF*, were selected and qRT-PCR analyses were performed for them on a test cDNA template [40]. The selection and testing of reference genes were developed according to a previously reported protocol [40]. The analysis of reference genes for the test samples was essential for statistical calculations because the expression of the reference gene was compared to the expression of the gene analyzed for each sample using the Gene Study tool (CFX Maestro from Bio-Rad Laboratories, Inc., Hercules, CA, USA).

### Preparation of candidate gene amplicons for Sanger sequencing

Sanger sequencing was performed to confirm that the desired amplicons were obtained for the candidate genes for *Lr46/Yr29* and to characterize the possible sequence polymorphism(s) among the resistant genotype (Glenlea cultivar) and the susceptible genotype (Artigas* cultivar). The majority of amplicons for sequencing were amplified from cDNA. However, *Lr46-RLK1*, *Lr46-RLK2*, and *Lr46-RLK4* were exceptions that resulted from obtaining a low quantity and/or quality PCR products using cDNA; in these cases, genomic DNA was used as template, isolated from leaves using the GeneMATRIX Plant and Fungi DNA Purification Kit (EURx Ltd, Gdańsk, Poland), according to the protocol provided by the manufacturer. For sequence confirmation of the analyzed candidate genes in the *Lr46*/*Yr29* locus, PCR reactions were performed for the respective primer pairs. GoTaq G2 Flexi DNA Polymerase reagents (Promega, Madison, WI, USA) were used to prepare the PCR reaction [40], otherwise identical in composition to qRT-PCR described above. The following amplification temperature profile was used: initial denaturation for 2 min at 94°C; followed by 35 cycles: denaturation for 1 min at 95 °C, annealing of primers for 30 s at 53.5 °C, extension for 30 s at 72 °C, extension for 30 s at 72 °C; final extension for 5 min at 72 °C and cooling to 4 °C. Purification of amplicons was performed using the QIAquick PCR Purification Kit (Qiagen Inc., Hilden, Germany), according to the protocol provided by the manufacturer. After purification, samples were resuspended in an elution buffer in a volume of 30 μL. Purified amplicons were directly sequenced using the Sanger method with the BigDye Terminator v3.1 Cycle Sequencing Kit (Applied Biosystems) and a 3730xl capillary DNA analyzer (Applied Biosystems) by Genomed (Warsaw, Poland). The results obtained were assembled using Geneious v8.1 software [47].

### Validation of miRNAs and analysis of their expression using stem-loop RT-PCR and ddPCR

In this study, the expression of four miRNAs was analyzed: three related to the candidate gene *Lr46-Glu2* (tae-miR5384-3p, tae-miR9780, tae-miR9775, tae-miR9780 also being complementary to *Lr46-RLK2*), and tae-miR164 complementary to *Lr46-RLK3*. The coding sequences of these candidate target genes downloaded from the Ensembl Plants database (https://plants.ensembl.org/Triticum_aestivum/Info/Index), were analyzed in the psRNATarget database (http://plantgrn.noble.org/psRNATarget/). The sequences of the four miRNAs analyzed were found in the miRBase database (https://www.mirbase.org/) and downloaded in FASTA format and using the Integrated DNA Technologies website (https://eu.idtdna.com/pages), stem-loop primers were designed for miRNA reverse transcription and ddPCR reactions according to the protocols [48–50]. The mirVana^™^ miRNA Isolation Kit with phenol (Thermo Fisher Scientific, Waltham, MA, USA) was used to extract the RNA fraction containing miRNA, the concentration and purity of which were measured spectrophotometrically using NanoDrop. The extracted miRNA fraction was then reversely transcribed using SuperScript IV Reverse Transcriptase (Thermo Fisher Scientific, Waltham, MA, USA), using stem-loop primers and the following protocol: incubation at 16 °C for 30 min; 60 cycles at 30 °C for 30 sec, 42 °C for 30 sec, and 50 °C for 1 sec; incubation at 85 °C for 5 min [51]. To quantify the number of miRNA molecules in the plant samples, a ddPCR mixture composed of 10 μL of ddPCR Super Mix Eva Green, primers (the final concentration of each primer was 200 nM), template (reversely transcribed, elongated miRNA), and RNase-free H2O was used. A 20 μL reaction mixture was used to generate the reaction droplets in an 8-well cartridge using a QX100 droplet generator (Bio-Rad Laboratories, Inc., Hercules, CA, USA). The droplets were carefully transferred to a 96-well ddPCR plate and heat-sealed with plastic covers with thermal foil (Bio-Rad Laboratories, Inc., Hercules, CA, USA). Then, cDNA was amplified in a T100 PCR thermal cycler (Bio-Rad Laboratories, Inc., Hercules, CA, USA) under the following cycling conditions: denaturation at 95 °C for 5 min, followed by 40 cycles with a three-step thermal profile of denaturation at 95 °C for 30 sec, annealing at 58 °C for 30 sec, and extension at 72 °C for 45 sec. Subsequently, the products were kept at 72 °C for 2 min for the final extension. After amplification, the products were cooled to 4 °C for 5 min and then heated to 90 °C for 5 min and finally cooled again to 12 °C. The droplets were quantified in a QX100 droplet reader (Bio-Rad Laboratories, Inc., Hercules, CA, USA). The data acquisition and analysis were conducted using QuantaSoft version 1.7 software (Bio-Rad Laboratories, Inc., Hercules, CA, USA). Positive droplets containing amplification products were distinguished from negative droplets by setting the fluorescence amplitude threshold to the lowest value of the positive droplet cluster. Yeast tRNA-Thr (TGT) molecules with the encoded sequence were added to each RT reaction as an internal control.

### Statistical analysis

The Kolmogorov–Smirnov test was used to evaluate the null hypothesis that a set of normalized expression data for a particular gene and cultivar comes from a normal distribution. To assess the homogeneity of variances, Bartlett’s test was used. Two-way analyses of variance (ANOVA) were carried out to determine the main effects of cultivar and time point as well as cultivar × time point interaction on the variability of the individual expression profiles of the analyzed genes of candidate genes for *Lr46/Yr29*. For comparison of means of expression in each examined time point after inoculation to expression before inoculation, the elementary contrasts were performed, conducted for each cultivar independently. The expression of the analyzed candidate genes is shown as heatmaps. Tukey’s Honestly Significant Difference tests (post-hoc factorial one- or two-way ANOVA, depending on ANOVA’s results) were performed in R v.4.4.0 [52] using package agricolae v.1.3–7 [53] at *α* = 0.05. We referred to the expression values of the candidate genes miRNAs analyzed as ’relative expression’ for the expression level, as our results represent differences in expression compared to the values before inoculation. For visualization of gene expression results, cluster analysis (UPGMA method) with Euclidean distances was performed. All these analyses were conducted using the GenStat statistical software package [54].

## Results

### Sanger sequencing results

**Fig 1:**
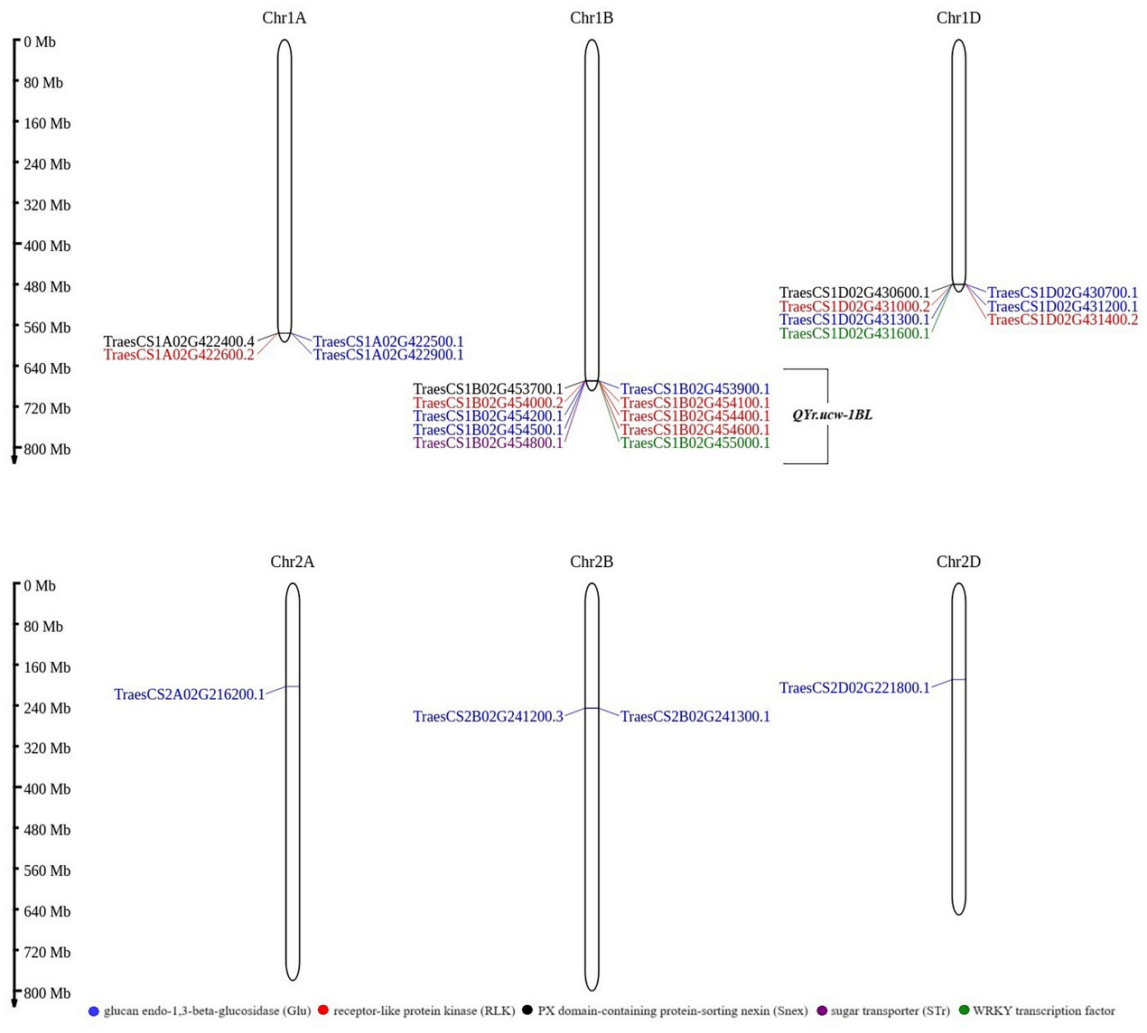
Localization of 10 candidate genes for *Lr46/Yr29* on chromosome 1B and homologues on wheat chromosomes 1A, 1D, 2A, 2B and 2D. Candidate genes for *Lr46/Yr29*, including the *QYr*.*ucw-1BL* region, have been highlighted by colors as per their biological functions/family identified in the legend (bottom). The selected candidate gene homologues were selected based on comparative analysis following Sanger sequencing of the obtained amplicons. The position of marker *csLV46G22* has also been indicated [29, 55]. ‘Ch1A’ and similar stand for the name of the chromosome and the abbreviation ‘Mb’ next to the scale stands for the Megabase unit referring to the physical length of the chromosomes. The genetic map was constructed using the MG2C_v2.1 tool [56].

The *Lr46/Yr29* region described in Cobo et al. [29] contains as many as four copies of the *RECEPTOR-LIKE PROTEIN KINASE* (*RLK*) gene and three copies of *GLUCAN ENDO-1*,*3-BETA-GLUCOSIDASES* (*Glu*) genes, and one copy each of the *SORTING NEXIN* (*Snex*) and the *WRKY TRANSCRIPTION FACTOR* (*WRKY*). The *RLK* and *Glu* genes occur in up to hundreds of copies throughout the wheat genome (https://www.ncbi.nlm.nih.gov/datasets/genome/GCF_018294505.1/, https://plants.ensembl.org/Triticum_aestivum/Info/Index). In addition, very similar gene arrangements are found on the two homoeologous chromosomes 1A and 1D in the same region as on 1B (Fig 1). Sequence similarity analysis of the *Lr46-Glu3* amplicon after Sanger sequencing also revealed the presence of copies on chromosomes 2A, 2B, and 2D. Therefore, a comparison was made of only those few copies of genes found in the region described, on chromosome 1B. These genes are highly differentiated, and it was not possible to design universal primers for them. Therefore, specific primers were designed, for each copy of the gene.

Analytical sequencing succeeded in obtaining the sequences for the majority of the target amplicons (Table 3). The full sequences of the obtained amplicons, location, and characterization of candidate genes for the *Lr46/Yr29* gene can be found in S1 File. For the first candidate, *Lr46-Glu1*, the TraesCS1B02G453900.1 sequence was amplified, but with a single nucleotide polymorphism (G) at position 40 compared with the TraesCS1A02G422500.1 sequence (G). For the ‘Glenlea’ cultivar, we observed a C+G at position 40, yielding a comparable level of amplification of TraesCS1B02G453900.1 and TraesCS1A02G422500.1. The *Lr46-Glu2* gene in the Artigas* cultivar amplified the TraesCS1A02G422900.1 sequence (with polymorphic A at position 38) and TraesCS1B02G454200.1 (with polymorphic G at position 38) (Fig 1). In the Glenlea cultivar, the exact TraesCS1A02G422900.1 homolog sequence was amplified (Fig 1). We failed to obtain a sequence that could be read correctly for *Lr46-RLK1* despite exhaustive attempts. For the *Lr46-RLK3*, the sequence of TraesCS1B02G454400.1 gene was amplified, with a small level of homolog TraesCS1D02G431000.1 (position 52) (Fig 1). The last candidate gene analyzed using the Sanger method was *Lr46-WRKY*, for which we obtained an amplicon (118 bp) of the TraesCS1D02G431600.1 gene, thus a homologue of the candidate gene TraesCS1B02G455000.1(Fig 1, Table 3, S1 File).

**Table 3:** Description of the resulting amplicons and candidate genes for *Lr46/Yr29* by Sanger sequencing.

Candidate gene abbreviation	Gene ID	Fragment length (bp)	Gene length (bp)	Amplified region (bp)	Gene symbol	UniProt accession
*Lr46-Glu1*	TraesCS1B02G453900.1	102	1722	308–410	LOC123100246	A0A3B5Z521
*Lr46-Glu2*	TraesCS1B02G454200.1	103	2529	677–779	LOC123148549	A0A3B5Z537
*Lr46-Glu3*	TraesCS1B02G454500.1	99	1968	635–733	LOC123100285	A0A3B5Z6G2
*Lr46-RLK1*	TraesCS1B02G454000.2	105	-	-	-	-
*Lr46-RLK2*	TraesCS1B02G454100.1	114	2526	1935–2047	LOC123148523	A0A3B5Z656
*Lr46-RLK3*	TraesCS1B02G454400.1	109	2217	1419–1527	LOC123100274	A0A3B5Z530
*Lr46-RLK4*	TraesCS1B02G454600.1	101	2514	1959–2041	LOC123148523	A0A3B5Z5H2
*Lr46-Snex*	TraesCS1B02G453700.1	103	3742	278–380	LOC123148452	A0A3B5Z651
*Lr46-WRKY*	TraesCS1D02G431600.1	118	1226	906–1024	LOC123100317	A0A3B6A1Y2

### Expression of candidate genes in analyzed varieties at different time-points (hpi)

**Fig 2:**
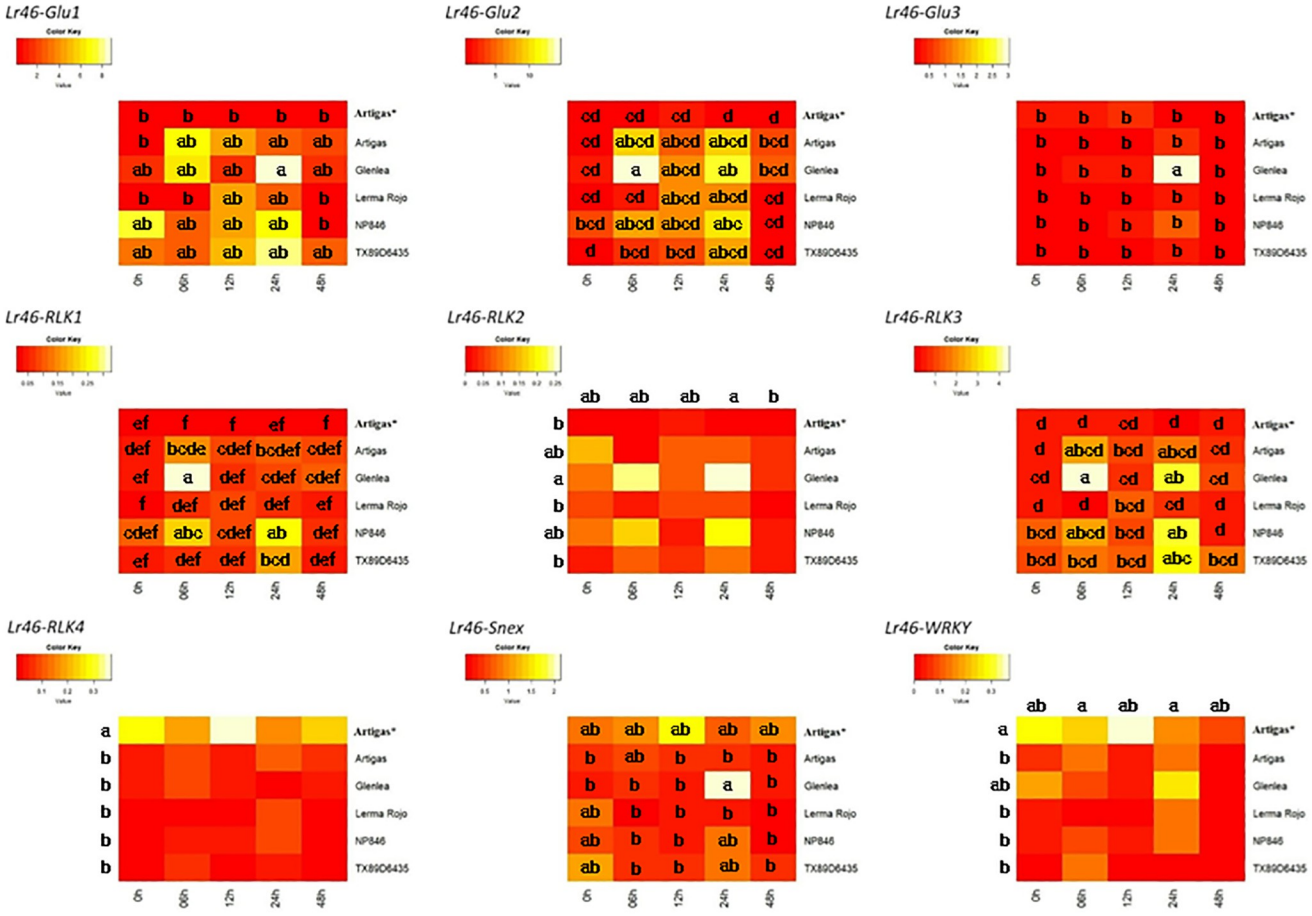
Expression heatmap of the nine *Lr46/Yr29* candidate genes in leaf tissues under *Puccinia triticina* infection. The graphs were generated based on the elemental contrasts carried out for each cultivar independently, and separately for each candidate gene. Tukey’s HSD test post ANOVA was carried out and the groups are shown on the heatmap as various letters, for statistically different groups at *α* = 0.05. Groups indicated on the heatmap denote significant interaction of cultivar × time point (hpi), whereas groups along horizontal axis (time points [hpi]) and/or vertical axis (cultivars) denote significance for single factors but not their interaction. The evaluation included reference cultivars of common wheat (Artigas*, Artigas, Glenlea, Lerma Rojo, TX89D6435, and NP846) indicated at the vertical axes at various time points (0, 6, 12, 24, and 48 hpi) identified at horizontal axes. Red indicates lower normalized expression and yellow—higher expression.

The qRT-PCR analyses of candidate genes in all studied wheat cultivars and at different time points are summarized as heatmap graphs (Fig 2). The raw data obtained from candidate gene expression analyses for *Lr46/Yr29* are presented in S1 Table. The elemental contrast values of the candidate genes tested are shown in S2 Table. Regarding the gene *Lr46-Glu1*, its expression varied greatly among the analyzed wheat cultivars as well as among the time points, therefore we did not observe any major common expression pattern (Fig 2). A decrease in the expression level of *Lr46-Glu2* gene was observed immediately after inoculation (6 hpi) for all cultivars except Glenlea. At 12 hpi, a further decrease in *Lr46-Glu2* expression levels was observed for all resistant cultivars (Fig 2). The inflection point occurred at 24 hpi when the defense response was activated for all cultivars. The expression level of *Lr46-Glu2* far exceeded the baseline level (0 hpi). At 48 hpi, the expression level of *Lr46-Glu2* decreased in all cultivars but still exceeded the baseline in Artigas, Glenlea, Lerma Rojo, and TX89D6435 cultivars. For the *Lr46-Glu3* gene, expression before as well as after inoculation was at very low levels (Fig 2). The only exception was the Glenlea cultivar at 24 hpi, where gene expression increased significantly. In the genes *Lr46-RLK1* and *Lr46-RLK2*, the observed expression increased at 6 hpi, shortly after inoculation, then decreased at 12 hpi, and we observed a renewed increase at 24 hpi. The highest expression levels at 6 hpi and 24 hpi, were observed in the NP846 cultivar and at 6 hpi in the ‘Glenlea’ (Fig 2). For *Lr46-RLK3*, the highest increases in expression after inoculation were observed in the cultivars TX89D6435 and NP946 at 24 hpi; whereas, in ‘Glenlea’ at 6 hpi and 24 hpi. However, this gene was expressed at relatively low levels (Fig 2). A special case is the expression of the *Lr46-RLK4* gene, whose highest expression values were observed in the Artigas* cultivar, in which, according to our team’s study, no molecular markers associated with the resistance conferred by *Lr34*, *Lr46*, or *Lr67* were identified [20, 40]. Expression of the *Lr46-RLK4* gene in the Artigas* cultivar was very high already before inoculation at 0 hpi (Fig 2). The *Lr46-Snex* gene showed low expression with two exceptions: at 12 hpi in the Artigas* cultivar and at 24 hpi in ‘Glenlea’ (Fig 2). In the *Lr46-WRKY* gene, we observed the highest values in ‘Artigas*’ at all time points except 48 hpi and in the Glenlea cultivar at 24 hpi (Fig 2). The qRT-PCR analyses of the *Lr46-STr* gene obtained many non-specific PCR products, which was particularly evident in the melt curve; as such, we decided to discard this gene’s data from further analyses (Table 2).

A two-way analysis of variance (ANOVA) was performed and identified the differences in the individual expression profiles of the analyzed genes for the cultivars at specific time points (Table 4). An additional source of variation was the cultivar × time point interaction. As each gene has a specific expression level, the analysis of variance was performed for each gene separately (Table 4). Cultivars exhibited unique patterns of gene expression in response to *Pt* infection. For example, ’Glenlea’ showed particularly high expression levels for several genes at 24 hpi, whereas ’Lerma Rojo’ exhibited relatively low expression levels across the majority of analyzed time points (S1 Table; Fig 2). For the majority of the *Lr46/Yr29* candidate genes, their expression significantly varied in time across the analyzed cultivars. Each gene responded differently to pathogen infection and varied in its expression levels across cultivars and time points. Notably, *Lr46-Glu2* consistently showed high expression levels across all cultivars and time points, indicating its potential importance in the defense response against *Pt*. For the *Lr46-Glu2* gene, we obtained comparably the highest values of mean squares compared to the other candidate genes (Table 4). The results obtained may thus suggest that the *Lr46-Glu2* gene harbors a special role in the *Pt* resistance response. In contrast, *Lr46-Snex* showed relatively low expression levels compared to other genes and exhibited variation across the analyzed time points but not among the cultivars. The analysis across the candidate genes for *Lr46*/*Yr29* revealed significant effects of both cultivar and time on gene expression in wheat. Notably, the susceptible cultivar Artigas* consistently exhibited lower expression levels compared to the resistant cultivars for various analyzed genes, which indicated its distinct gene expression profile under the *Pt* stress. For several analyzed genes, including *Lr46*-*Glu1* and *Lr46*-*Glu2*, significant interactions between cultivar and time underscored the dynamic nature of gene expression in response to temporal factors and genetic variation. Post-hoc analysis further elucidated these findings and revealed distinct expression patterns among wheat cultivars across different time points (Fig 2). Resistant cultivars often exhibited comparatively higher expression levels compared to the susceptible control cultivar, thus emphasizing their potential role in mediating wheat’s defense response against *Pt*.

**Table 4:** Mean squares from two-way analysis of variance (ANOVA) for the individual expression profiles of the analyzed genes of candidate genes for *Lr46/Yr29*.

Source of variation	Cultivar	Time point	Cultivar × time point interaction	Residual DF	Residual
Degrees of freedom (DF)	5	4	20	(variable)	50
*Lr46-Glu1*	38.87 ***	34.65***	13.98*	59	6.35
*Lr46-Glu2*	93.29***	126.35***	17.02*	60	9.3
*Lr46-Glu3*	0.8425**	1.791***	0.717***	60	0.24
*Lr46-RLK1*	0.031***	0.028***	0.009***	59	0.002
*Lr46-RLK2*	0.034***	0.025***	0.006^ns^	52	0.004
*Lr46-RLK3*	6.629***	8.579***	2.164***	59	0.746
*Lr46-RLK4*	0.106***	0.002^ns^	0.006^ns^	57	0.009
*Lr46-Snex*	0.913**	0.682*	0.478*	60	0.26
*Lr46-WRKY*	0.082***	0.035*	0.011^ns^	48	0.01

*, **, ***–significant at 0.05, 0.01, 0.001 levels, respectively;

ns–not significant

**Fig 3:**
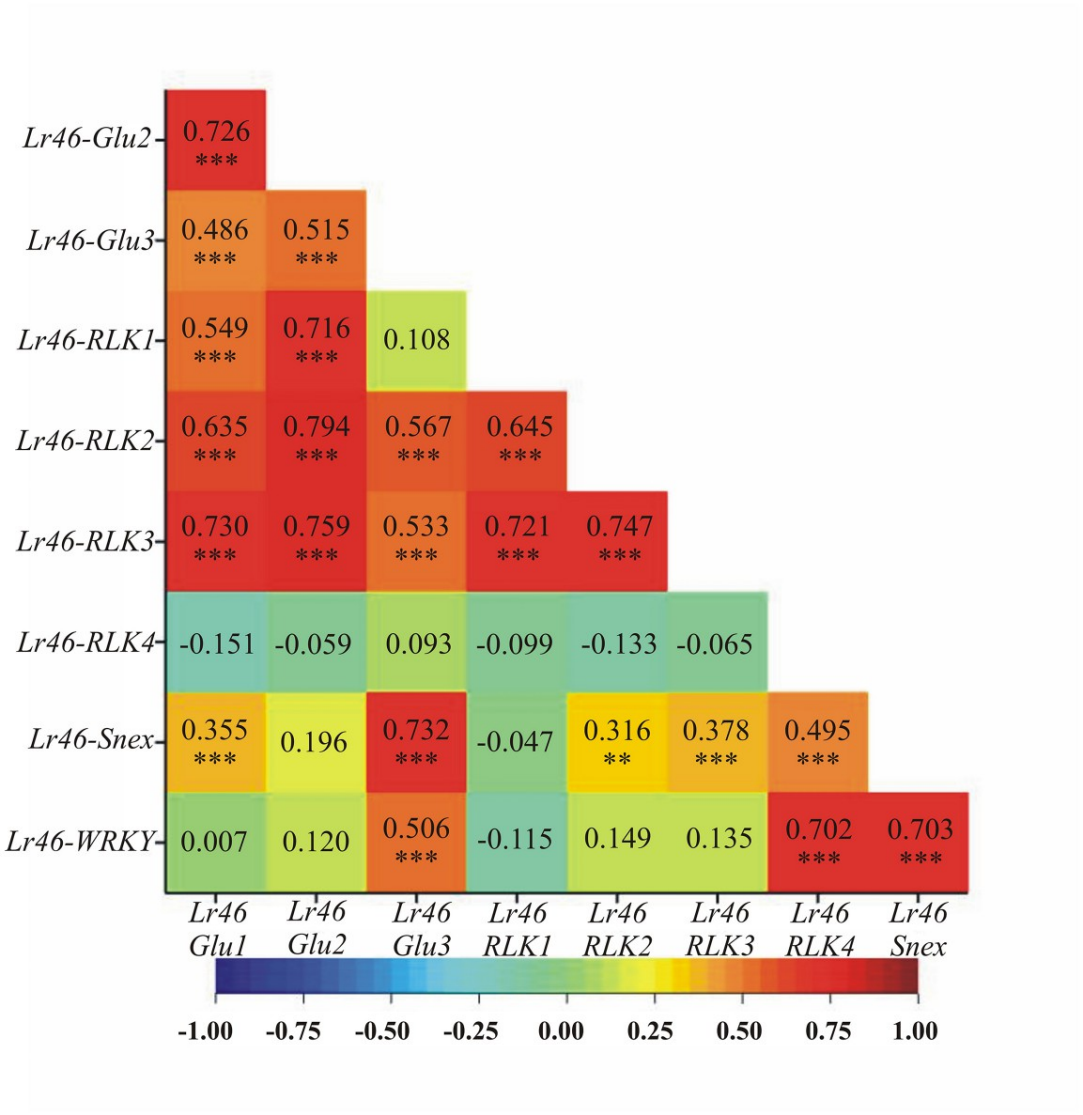
Heat maps of Pearson’s linear pairwise correlation coefficients between the observed candidate genes for *Lr46*/*Yr29* based on the values of their individual expression profiles. Significance of pairwise correlations is marked: * *p* < 0.05, ** *p* < 0.01, *** *p* < 0.001. Heatmap color scheme (labeled at the bottom) informs of more positive (red hue) or negative (blue hue) correlations.

A comparison of expression profiles between genes was also attempted by correlation analysis (Fig 3). Among the candidate genes tested, the highest positive correlation was observed for the expression profile between candidate genes: *Lr46-Glu2* and *Lr46-RLK2* (0.794; *p* < 0.001) (Fig 3). There was also a positive correlation for the *Lr46-Glu1* gene between the genes: *Lr46-Glu2*, *Lr46-RLK1*, *Lr46-RLK2* and *Lr46-RLK3*, where there were high correlations between the *Lr46-Glu1* gene and the genes: *Lr46-Glu2* and *Lr46-RLK3* (0.726 and 0.730, respectively; *p* < 0.001) (Fig 3). The *Lr46-Glu2* gene showed positive correlations (*p* < 0.001) with the other candidate genes tested except for genes: *Lr46-RLK4*, *Lr46-Snex*, and *Lr46-WRKY*, where the correlation was close to 0 with *p* > 0.05 (Fig 3). The *Lr46-Glu3* gene showed a positive and statistically significant correlations with the majority of candidate genes except *Lr46-RLK1* and *Lr46-RLK2* (0.108 and 0.093, respectively, not statistically significant). Interestingly, for the genes: *Lr46-RLK1*, *Lr46-RLK2*, and *Lr46-RLK3*, we observed a general presence of correlation with *Glu* or *RLK* candidate genes, but general absence of correlation with other candidate genes (Fig 3). However, a distinct trend was observed for *Lr46-RLK4*, where we observed correlations with only two genes: *Lr46-Snex* and *Lr46-WRKY* (0.495 and 0.702, respectively; *p* < 0.001). Interestingly, there was a tendency between the *Lr46-RLK4*, *Lr46-Snex*, and *Lr46-WKRY* genes to correlate with each other (Fig 3). The exception is *Lr46-Snex*, where there is a high correlation also between the *Lr46-Glu3* gene (0.735, *p* < 0.001).

### Expression of miRNAs complementary to target candidate genes

**Fig 4:**
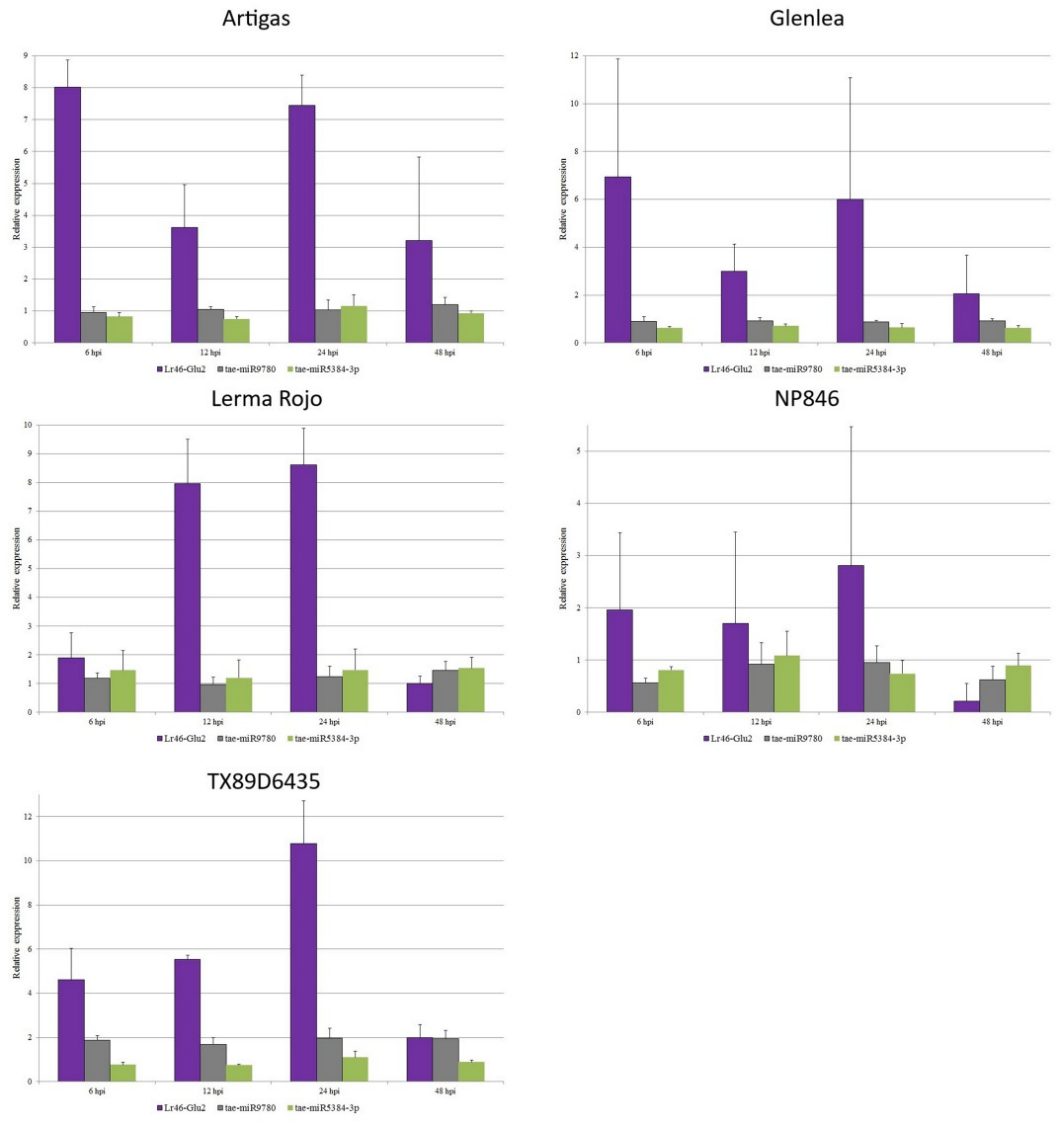
Analysis of complementary miRNAs expression compared to *Lr46-Glu2* expression patterns during *Puccinia triticina* infection. The numbers 6, 12, 24, and 48 stand for hours post inoculation (hpi). The graphs show relative expression values compared to the 0 hpi time point. The error bars indicate the standard error (SE). Data were obtained from the average of three biological replicates, and each biological replicate had three technical replicates, according to the MIQUE protocol.

**Fig 5:**
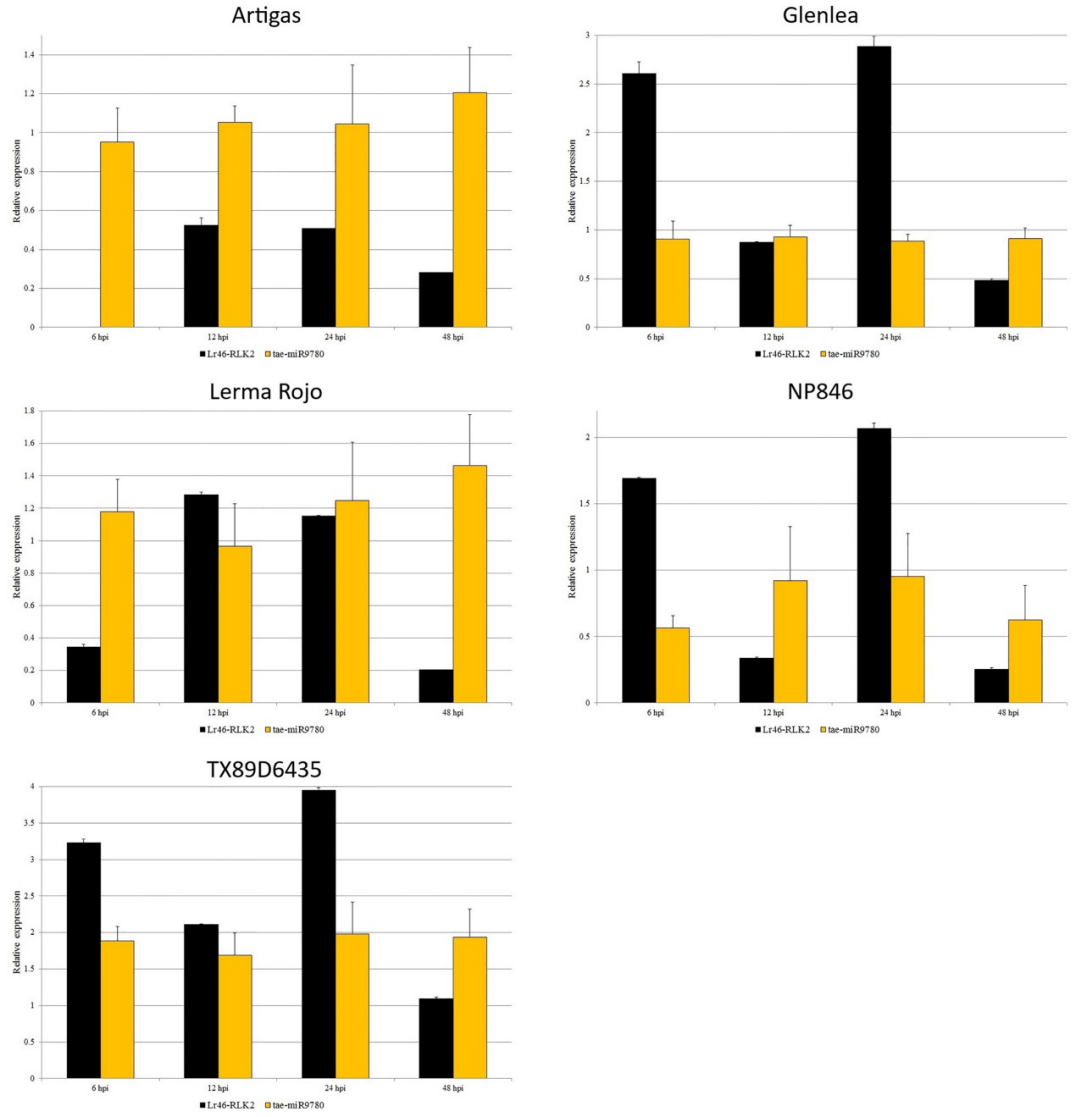
Analysis of complementary miRNA expression compared to *Lr46-RLK2* expression patterns during *Puccinia triticina* infection. The numbers 6, 12, 24 and 48 stand for hours post inoculation (hpi). The graphs show relative expression values compared to the 0 hpi time point. The error bars indicate the standard error (SE). Data were obtained from the average of three biological replicates, and each biological replicate had three technical replicates, according to the MIQUE protocol.

**Fig 6:**
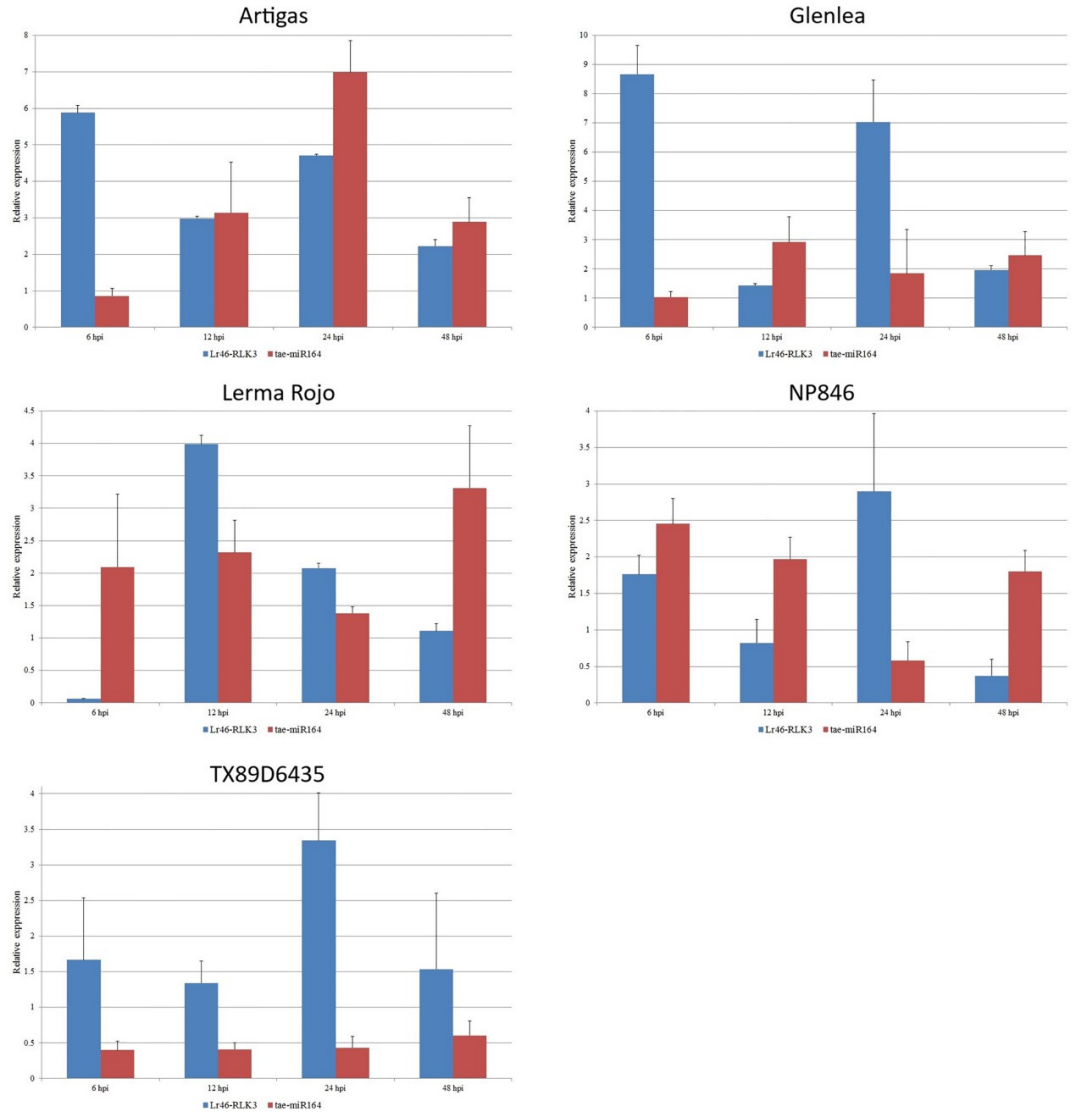
Analysis of complementary miRNA expression compared to *Lr46-RLK3* expression patterns during *Puccinia triticina* infection. The numbers 6, 12, 24 and 48 stand for hours post inoculation (hpi). The graphs show relative expression values compared to the 0 hpi time point. The error bars indicate the standard error (SE). Data were obtained from the average of three biological replicates, and each biological replicate had three technical replicates, according to the MIQUE protocol.

Using stem-loop RT-PCR and ddPCR, we performed expression analysis of miRNAs selected from databases as complementary to several candidate genes for *Lr46/Yr29*. Contrast analysis was performed for the expression data of miRNAs, and the resulting elemental contrast values for the expression of miRNAs complementary to the candidate genes can be found in S3 Table. We compared the expression results of the candidate genes with the respective values for the miRNAs complementary to them. According to the databases, tae-miR9780 is complementary to two candidate genes, *Lr46-Glu2* (TraesCS1B02G454200.1) and *Lr46-RLK2* (TraesCS1B02G454100.1). Towards the *Lr46-Glu2* gene, tae-miR9775 is also complementary. Unfortunately, the expression of tae-miR9775 was at a low level and did not allow for confident inferences. In the *Lr46-Glu2* gene, we analyzed the expression of two miRNAs: tae-miR9780 and tae-miR5384-3p. The expression of tae-miR9780 in the majority of the tested wheat cultivars was at a constant level, both before and after inoculation. The exceptions were the TX89D6435 and Lerma Rojo cultivars where values higher than at 0 hpi were observed (Fig 4). Especially for the TX89D6435 cultivar, the results suggested an up-regulation. The tae-miR5384-3p expression showed fairly constant expression levels at all time points. Here, again in the Lerma Rojo cultivar, the miRNA was expressed with values higher than at 0 hpi. At 6 hpi, expression increased with the *Lr46-Glu2* gene, whereas it decreased slightly at 12 hpi with a large increase in its complementary candidate gene expression. On the other hand, at 24 hpi, tae-miR5384-3p expression increased again together with an increase in its complementary candidate gene, whereas at 48 hpi we observed a large decrease in *Lr46-Glu2* expression together with an increase in tae-miR5384-3p (Fig 4). In the case *Lr46-RLK2*, to which tae-miR9780 is also complementary, relationships similar to those of *Lr46-Glu2* were observed. The expression of both *Lr46-RLK2* and tae-miR9780 showed very low values (Fig 5). In contrast, tae-miR164, which is complementary to the candidate gene *Lr46-RLK3* (TraesCS1B02G454400.1), showed differential expression at pre- and post-inoculation. The expression of the *Lr46-RLK3* gene and its complementary tae-miR164 is shown in Fig 6 and suggests that tae-miR164 is a molecule that may be down-regulating its target gene’s expression. In the majority of the tested wheat cultivars, when candidate gene expression increased, then tae-miR164 was down-regulated, and vice versa: when miRNA expression increased then candidate gene expression was down-regulated (Fig 6).

## Discussion

In this study, we attempted to analyze the expression of candidate genes for the *Lr46/Yr29* locus—selected region of *QYr*.*ucw-1BL* [29]—after inoculation with the fungus *P*. *triticina* under controlled conditions. Our research hypothesis recognizes that a single causal gene is responsible for the desirable APR-type *Pt* resistance; however, we cannot exclude the alternative hypothesis of a cluster of closely linked resistance genes, each effective against different pathogen(s). Although cloning of these genes would be necessary to provide a conclusive test for this hypothesis, determining the ability of the *QYr*.*ucw-1BL* region to confer APR against leaf rust, stem rust, and powdery mildew pathogens could provide valuable information to experimentally validate this hypothesis. We functionally examined the candidate genes and most of them were potentially related to plant defense, making it difficult to identify the actual causal gene for *Lr46/Yr29/Sr58*. We were able to analyze the expression profiles for nine out of ten candidate genes encompassed by that locus at five time points (0, 6, 12, 24, and 48 hpi): TraesCS1B02G453900.1, TraesCS1B02G454200.1, TraesCS1B02G454500.1, TraesCS1B02G454000.2, TraesCS1B02G454100.1, TraesCS1B02G454400.1, TraesCS1B02G454600.1, TraesCS1B02G453700.1, and TraesCS1B02G455000.1. Consistent with our research assumption, the highest expression of *Lr46/Yr29* candidate genes occurred at 12 and 24 hpi, as confirmed by literature data for the *Lr* genes [57, 58]. Such an expression profile was obtained for only one candidate gene among the nine studied candidate genes (TraesCS1B02G454200.1; gene abbreviation *Lr46-Glu2*), which may suggest its involvement in the immune response mechanisms to *Pt* infection. Future studies will address this gene’s functional validation by gene knockout or overexpression studies, to confirm its role in *Pt* resistance. Similarly, future integration of transcriptomic, proteomic, and metabolomic data could provide a more comprehensive understanding of the molecular mechanisms underlying resistance to *Pt*. Multi-omics approaches enable the identification of key pathways and regulatory networks involved in the defense response, offering novel targets for genetic improvement [59]. Our study explored the expression patterns across various wheat cultivars, whereas their genetic background and specific resistance mechanisms are not thoroughly known. Investigating the genetic diversity and evolutionary history of these cultivars could provide context for interpreting the observed gene expression patterns. For example, future investigations of the genomic context and copy number variation of candidate genes could elucidate their evolutionary history and functional significance in relation to resistance or other desirable phenotypes.

The study by Prasad et al. [5] meta-analyzed 298 major scientific reports on mechanisms of plant resistance to fungal pathogens, including *Pt*. The authors indicated that the continued search for diverse and durable resistance to *Pt* must be pursued using advanced tools and techniques, including molecular methods. Understanding the molecular basis of plant-pathogen interactions will enable the development of new strategies for *Pt* resistance in common wheat. To date, information on the cloning of the following *Lr* genes has been reported in the literature: *Lr1*, *Lr10*, *Lr21*, *Lr22a*, *Lr34/Yr18/Pm38/Sr57*, and *Lr67/Yr46/Pm46/Sr55* [5, 21, 60–63]. Despite tangible progress in comprehending the molecular basis of *Pt* resistance, the role of miRNAs in modulating gene expression has only been studied for a few major R genes, which included *Lr24*, *Lr28*, and *Sr24* [35, 39, 64, 65]. miRNAs have been shown to perform important roles as negative regulators through repression of translation or degradation of host and/or pathogen target mRNAs [32, 66]. In common wheat, although the role of miRNAs in pathogen resistance has been analyzed, including resistance to *Pt* involving the *Lr46/Yr29* gene [50], very few similar studies have been conducted for genes that confer the APR-type of resistance.

The qRT-PCR method is an irreplaceable tool for the amplification, identification, and quantification of nucleic acids. The technique is distinguished by its ability to detect genes and measure gene expression quantitatively and specifically. qRT-PCR is a valued method for both research and diagnostic applications. Cobo et al. [29] highlighted the need to examine the expression in different host plant genotypes with and without pathogen inoculation to determine whether there are differences in candidate gene expression associated with differences in immunity. In the presented study, we report the results of candidate gene expression analyses for *Lr46/Yr29*. Except for the *Lr46-RLK2*, *Lr46-RLK4*, and *Lr46-WRKY* genes, ANOVA indicated interactions in the expression profiles for the remaining six genes between the analyzed wheat cultivars and the time points. In the candidate gene region, three genes have been identified as *GLUCAN ENDO-1*,*3-BETA-GLUCOSIDASES* (TraesCS1B02G453900.1, TraesCS1B02G454200.1, TraesCS1B02G454500.1) [29]. These genes are found in many seed plant species and are involved in plant defense against pathogens, and their expression is often dramatically induced when plants are infected by fungal, bacterial, or viral pathogens [29, 67]. Glucan endo-1,3-β-glucosidases (β-1,3-glucanases) are one of the important hydrolytic enzymes among the pathogenesis-related proteins (PR proteins) that are abundant in many plant species after infection with different types of pathogens [68, 69]. A single plant species may have various homologues of the glucano-1,3-β-glucosidase genes, important for retarding the growth of pathogenic fungi by the decomposition of their cell walls which contain β-glucans. Glucan endo-1,3-β-glucosidase appears to be overexpressed together with chitinases after fungal infection. The induction of these two hydrolytic enzymes has been described in a number of plant species, including pea, bean, tomato, tobacco, maize, soybean, potato, and wheat [67–72]. Notably, an increase in levels of glucan endo-1,3-β-glucosidase was observed during drought stress [73].

Many *RECEPTOR-LIKE PROTEIN KINASE* (*RLK*) gene families can be found in plants. RLKs are membrane proteins located in the extracellular domain of the plant reception networks and are involved in both biotic and abiotic stress responses. The extracellular ligand-binding domain, the transmembrane domain, and the intracellular protein kinase domain are typical components of RLKs. The extracellular domain, which is specific to individual RLKs, binds a particular ligand and allows RLKs to respond to different types of signals. Among the RLKs, variants such as leucine-rich repeats, lectin, lysine motif, or wall-associated kinases are distinguished [74, 75]. RLKs make up the largest known family of protein kinases that are important in the plant response to pathogen infection. Gu et al. [74] discovered a new cysteine-rich RLK gene, *TaCRK2*, which positively regulated resistance to infection caused by *Pt* in wheat. In our study, four of the candidate genes are RLKs (*Lr46-RLK1*, *Lr46-RLK2*, *Lr46-RLK3*, and *Lr46-RLK4*) and encode proteins important for the recognition of extracellular signals and the initiation of intracellular signaling cascades in reaction to those stimuli [76]. The RLKs in the *Lr46/Yr29* locus encode proteins with two extracellular domains that are characteristic of the subgroup of *CYSTEINE-RICH RECEPTOR KINASES*. [29]. It has been proposed that members of this sub-group may be involved in redox signaling [77]. In a recent study [78], researchers investigated differential gene expression under *Pt* stress, which was analyzed in a close isogenic line carrying the leaf rust resistance gene *Lr57*. The highest number of transcripts undergoing expression was detected at 12 hpi. Interestingly, among these transcripts, a cysteine-rich RLKs expressed only in the resistant genotypes at 12 hpi.

The WRKY family represents another group of transcription factors important in regulating plant responses to stress factors. In *Arabidospis*, overexpression of *WRKY63/ABO3* [79] and *WRKY57* [80] resulted in increased drought stress tolerance in an ABA-dependent manner. Numerous data indicate that overexpression of genes encoding proteins belonging to the WRKY family also increases dehydration tolerance in crop species [81–83]. Many research papers have described WRKY gene family in crops, including rice (*Oryza sativa*), maize (*Zea mays*), sorghum (*Sorghum bicolor*), and cabbage (*Brassica rapa*) [76, 84, 85].

Another method that allows expression analysis is ddPCR (droplet digital PCR), referred to as the third-generation PCR method. This technique is characterized by high sensitivity and precision in amplicon detection. The system exclusively reads the target molecules as positive and all others as negative, based on which the number of target molecules in the sample is calculated, without the need to refer to standard curves or a reference gene (in contrast to the qRT-PCR method) [86]. In our work, ddPCR was used to analyze the expression of miRNA molecules: tae-miR5384-3p, tae-miR9780, tae-miR9775, and tae-miR164. Previous sequence analysis revealed the involvement of tae-miR5384-3p (as well as tae-miR164 and tae-miR9679-5p) in targeting the *TaAFB6* gene (TraesCS5A02G281100), which encodes the AUXIN SIGNALING F-BOX (AFB) protein [87]. This suggested the possibility of post-transcriptional regulation of *TaAFB*-type gene expression. In that study, researchers analyzed the expression of genes of selected AFBs for this purpose by carrying out inoculation with spores of *Pt* taking wheat leaf fragments at five time points (0, 12, 48, 120, and 168 hpi) [87]. The fraction of *AFB* genes analyzed showed the highest expression at 12 and 48 hpi in both susceptible and resistant isogenic wheat lines. All known *TaAFB-*type genes are involved in the auxin-activated signaling pathway that results in the ubiquitination process [87].

The tae-miR5384-3p can inhibit a number of genes in the TaFAB2 subfamily, which are associated with the formation of unsaturated fatty acids that play an important role in plant development and in response to biotic and abiotic stresses [88]. In contrast, tae-miR5384-3p showed a very high increase in expression after treating wheat seedlings with a chitosan suspension [89]. The type of fluctuating relative expression profiles is commonly observed for small RNA sequencing or qPCR-based miRNA expression analysis under different biotic and abiotic stress conditions [90]. Such a phenomenon may relate to changing defense mechanisms in the early and late stages of the plant response. A recent study [91] used computational methods to investigate the interaction between the wheat miRNA transcriptome and mosaic viruses in wheat. In wheat streak mosaic virus (WSMV) and wheat mosaic virus (TriMV) infections, three miRNAs (including tae-miR5384-3p) were among the most up-regulated miRNAs. This may suggest the involvement of this miRNA in the overall immune response in wheat.

The miR164 family is one of the most conserved groups of miRNAs in plants [92]. One miRNA molecule can control multiple target genes, similar to plant transcription factors. Previous studies have shown that miR164 targets plant-specific NAC transcription factors [93]. The majority of NACs play an essential role in the regulation of plant development and the response to abiotic and biotic stresses. The functions of miR164 have been associated with plant responses to biotic stresses. The main function attributed to miR164 is to regulate transcript levels of relevant genes [92, 94, 95]. This miRNA also targets *Lr46-RLK3* candidate gene. In our study, in the majority of tested wheat cultivars, when candidate gene expression increased, tae-miR164 was down-regulated, and vice versa: when miRNA expression increased, the candidate gene expression values were down-regulated. The decrease in tae-miR164 may be related to the activation of *Lr46-RLK3* gene-dependent immune mechanisms, observed as a rapid gene response to the pathogen. Moreover, we observed similar relationships in our previous work on tae-miR164 [50]. Exploring the regulatory networks involving miRNAs and their target genes in the future could uncover additional layers of complexity in the defense responses of wheat to *Pt*.

## Conclusions

In this study, we tracked down the desirable APR-type *Pt* resistance in wheat conferred by a single gene(s). Our qRT-PCR experiment successfully studied the expression of nine analyzed candidate genes encompassed by the *Lr46/Yr29* locus, except the candidate gene *Lr46-STr*. Among these genes, the least differential expression profile was observed for the candidate gene *Lr46-Glu2*, with an increase in its expression after 24 hours, which may suggest activation of the defense response in all resistant cultivars. The expression level of *Lr46-Glu2* far exceeded the pre-inoculation baseline. After 48 hpi, the expression level of *Lr46-Glu2* declined in all cultivars but in cultivars Artigas, Glenlea, Lerma Rojo, and TX89D6435 it still exceeded the pre-inoculation baseline. The possibility that the expression of the candidate gene *Lr46-Glu2* was controlled by tae-miR5384-3p was observed: For the cultivars Glenlea and NP846 at all time points at which the expression of this gene increases, the expression of its complementary tae-miR5384-3p decreases. Conversely, when the expression of *Lr46-Glu2* decreased, the expression of tae-miR5384-3p increased. For other cultivars, similar patterns were observed only at specific time points: Artigas (6 hpi), Lerma Rojo (12 hpi), and TX89D6435 (6 hpi and 12 hpi). Our research highlights the need to understand the molecular basis of plant-pathogen interactions, and this will enable the development of new strategies for *Pt* resistance. Cloning and functional analyses of the *Lr46/Yr29* locus are essential to determine the allelic variation of the gene(s) and to establish their function(s). Obtaining more information and knowing the gene sequence that confers *Pt* resistance would enable the development of more specific molecular markers for *Lr46/Yr29*, which would facilitate line selection in wheat resistance breeding.

## Supporting information

S1 File Characterization of the obtained amplicons (candidate genes for *Lr46/Yr29*) by Sanger sequencing. (PDF)

S1 Table Expression of the nine *Lr46/Yr29* candidate genes in leaf tissues under *Puccinia triticina* infection. (PDF)

S2 Table Elemental contrast values for candidate genes expression. The table shows the elemental contrasts for each cultivar independently in order to compare mean expression at each time point tested after inoculation to expression before inoculation.
(PDF)

S3 Table Elemental contrast values for expression of miRNA molecules complementary to candidate genes. The table shows the elemental contrasts for each cultivar independently to compare the average expression at each time point tested after inoculation with the expression before inoculation.
(PDF)
