# Supplementary material for: Expression patterns of candidate genes for the Lr46/Yr29 “slow rust” locus in common wheat (Triticum aestivum L.) and associated miRNAs inform of the gene conferring the Puccinia triticina resistance trait
Source: PLoS One. 2024 Sep 6;19(9):e0309944. doi: 10.1371/journal.pone.0309944 (PMC11379320; doi:10.1371/journal.pone.0309944)
Supplement: S1 File — (PDF) [file pone.0309944.s001.pdf]

Supplementary Materials S1. Description of the obtained amplicons (candidate genes for *Lr46/Yr29*) by Sanger sequencing.

1. **TraesCS1B02G453900.1 (*Lr46-Glu1*) - partial CDS**
   SEQUENCE ID: XM_044522204.1
   GENE SYMBOL:LOC123100246
   SOURCE: Triticum aestivum (bread wheat)
   ORGANISM: Triticum aestivum
   Eukaryota; Viridiplantae; Streptophyta; Embryophyta; Tracheophyta; Spermatophyta; Magnoliopsida; Liliopsida; Poales; Poaceae; BOP clade; Pooideae; Triticodae; Triticeae; Triticinae; Triticum.
   CHROMOSOME: 1B
   GENE LENGTH: 1722 bp
   PROTEIN: glucan endo-1,3-beta-D-glucosidase
   BIOLOGICAL PROCESS: carbohydrate metabolism process
   UniProt ACCESSION: A0A3B5Z521
   CULTIVAR: Artigas (PI 73046); Glenlea (CItr 17272)
   TEMPLATE: mRNA (cDNA)
   FRAGMENT LENGTH: 102 bp
   GENE REGION: 308 – 410 bp
   REFERENCE GENOME: Triticum aestivum (taxid: 4565)
   CDS sequence:
   GGTGCAGAGCAATGTGAAGGCCTACTACCCGGCCACGCTGATCAATGGCGTGACGGTC
   GGGAACGAGGTGTTCAAAGAGGCTAGCCAACTAAACTCTCAGCT

2. **TraesCS1B02G454200.1 (*Lr46-Glu2*) - partial CDS**
   SEQUENCE ID: XM_044567989.1
   GENE SYMBOL: LOC123148549
   SOURCE: Triticum aestivum (bread wheat)
   ORGANISM: Triticum aestivum
   Eukaryota; Viridiplantae; Streptophyta; Embryophyta; Tracheophyta; Spermatophyta; Magnoliopsida; Liliopsida; Poales; Poaceae; BOP clade; Pooideae; Triticodae; Triticeae; Triticinae; Triticum.
   CHROMOSOME: 1B
   GENE LENGTH: 2529 bp
   PROTEIN: glucan endo-1,3-beta-D-glucosidase
   BIOLOGICAL PROCESS: carbohydrate metabolism process
   UniProt ACCESSION: A0A3B5Z537
   CULTIVAR: Artigas (PI 73046)
   TEMPLATE: mRNA (cDNA)
   FRAGMENT LENGTH: 103 bp
   GENE REGION: 677 – 779 bp
   REFERENCE GENOME: Triticum aestivum (taxid: 4565)
   CDS sequence:
   TATCTCTTGTTCCGCCCCAACGCTGGCATTCTTGACAGGAATAGCAGGCAAACGTACTT
   CAGCCTCCTCGACGCGCAGCTTGACGCTGTGTACTATGCGATGG

3. TraesCS1B02G454500.1 (*Lr46-Glu3*) - partial CDS

SEQUENCE ID: XM_044522234.1
GENE SYMBOL: LOC123100285
SOURCE: Triticum aestivum (bread wheat)
ORGANISM: Triticum aestivum
Eukaryota; Viridiplantae; Streptophyta; Embryophyta; Tracheophyta; Spermatophyta; Magnoliopsida; Liliopsida; Poales; Poaceae; BOP clade; Pooideae; Triticodae; Triticeae; Triticinae; Triticum.
CHROMOSOME: 1B
GENE LENGTH: 1968 bp
PROTEIN: glucan endo-1,3-beta-D-glucosidase
BIOLOGICAL PROCESS: carbohydrate metabolism process
UniProt ACCESSION: A0A3B5Z6G2
CULTIVAR: Artigas (PI 73046)
TEMPLATE: mRNA (cDNA)
FRAGMENT LENGTH: 99 bp
GENE REGION: 635 – 733 bp
REFERENCE GENOME: Triticum aestivum (taxid: 4565)
CDS sequence:
ACTCCAGACGTCATTCCCGCCATCCGCCGGCGCCTTCAAGGACGACATCGCGCTGTCA
GTGATGAGTCCCATGCTCGACTTTTTCCGACAGACCGGTTC

4. TraesCS1B02G454100.1 (*Lr46-RLK2*) - partial CDS with intron

SEQUENCE ID: XM_044567961.1
GENE SYMBOL: LOC123148523
SOURCE: Triticum aestivum (bread wheat)
ORGANISM: Triticum aestivum
Eukaryota; Viridiplantae; Streptophyta; Embryophyta; Tracheophyta; Spermatophyta; Magnoliopsida; Liliopsida; Poales; Poaceae; BOP clade; Pooideae; Triticodae; Triticeae; Triticinae; Triticum.
CHROMOSOME: 1B
GENE LENGTH: 2526 bp
PROTEIN: Cysteine-rich receptor-like protein kinase
BIOLOGICAL PROCESS: ATP binding, protein kinase activity
UniProt ACCESSION: A0A3B5Z656
CULTIVAR: Glenlea (CItr 17272)
TEMPLATE: gDNA
FRAGMENT LENGTH: 113 bp (190 bp with intron)
GENE REGION: 1935 – 2047 bp
REFERENCE GENOME: Triticum aestivum (taxid: 4565)
Sequence:
TGAGATCGTGACGGGAAGGAAGAACACTGACAGCTACAACTCCCAACAATCTCAGGA
TCTCCTGACGACCGTATGTGCTTGTTATCTGAATGTCTGACTAACTAACCTGCAGTGCA
GCTTGGCTCATCGATCCTACAAATCGTGCAGGTATGGGAGCATTGGACGGCCGGAACA
CTACTGGAGATGCTAG

5. **TraesCS1B02G454400.1 (*Lr46-RLK3*) - partial CDS**
SEQUENCE ID: XM_044522224.1
GENE SYMBOL: LOC123100274
SOURCE: Triticum aestivum (bread wheat)
ORGANISM: Triticum aestivum
Eukaryota; Viridiplantae; Streptophyta; Embryophyta; Tracheophyta; Spermatophyta; Magnoliopsida; Liliopsida; Poales; Poaceae; BOP clade; Pooideae; Triticodae; Triticeae; Triticinae; Triticum.
CHROMOSOME: 1B
GENE LENGTH: 2217 bp
PROTEIN: Cysteine-rich receptor-like protein kinase
BIOLOGICAL PROCESS: ATP binding, protein kinase activity
UniProt ACCESSION: A0A3B5Z530
CULTIVAR: Artigas (PI 73046); Glenlea (CItr 17272)
TEMPLATE: mRNA (cDNA)
FRAGMENT LENGTH: 109 bp
GENE REGION: 1419 – 1527 bp
REFERENCE GENOME: Triticum aestivum (taxid: 4565)
CDS sequence:
CAGGGACCTTAAAGCTAATAATATTCTTCTTGACGAGGAGATGGATCCTAAAATCGCAG
ACTTTGGATTGGCAAGGCTGCTACAAGAAGGTCACACTCATACTCAAACC

6. **TraesCS1B02G454600.1 (*Lr46-RLK4*) - partial CDS with intron**
SEQUENCE ID: XM_044567973.1
GENE SYMBOL: LOC123148523
SOURCE: Triticum aestivum (bread wheat)
ORGANISM: Triticum aestivum
Eukaryota; Viridiplantae; Streptophyta; Embryophyta; Tracheophyta; Spermatophyta; Magnoliopsida; Liliopsida; Poales; Poaceae; BOP clade; Pooideae; Triticodae; Triticeae; Triticinae; Triticum.
CHROMOSOME: 1B
GENE LENGTH: 2514 bp
PROTEIN: Cysteine-rich receptor-like protein kinase
BIOLOGICAL PROCESS: ATP binding, protein kinase activity
UniProt ACCESSION: A0A3B5Z5H2
CULTIVAR: Artigas (PI 73046); Glenlea (CItr 17272)
TEMPLATE: gDNA
FRAGMENT LENGTH: 101 bp (185 bp with intron)
GENE REGION: 1959 – 2041 bp
REFERENCE GENOME: Triticum aestivum (taxid: 4565)
Sequence:
TTCAGCTTTGGCGTATTGCAACTCCCAACAATCTCAGGATCTCCTGACGACCGTACGTG
CTTGTTATCTGAATGTCTGACTAACTAACCTGCAGGCTGCAGTGCAGTTTGGCTCATCG
ATCCTACAAATCGTGCAGGTATGGGAGCATTGGACGGCTGGAACAGTACTGGAGATGC
TAGACCCGT

7. **TraesCS1B02G453700.1 (*Lr46-Snex*) - partial CDS**
   SEQUENCE ID: XM_044567888.1
   GENE SYMBOL: LOC123148452
   SOURCE: Triticum aestivum (bread wheat)
   ORGANISM: Triticum aestivum
   Eukaryota; Viridiplantae; Streptophyta; Embryophyta; Tracheophyta; Spermatophyta; Magnoliopsida; Liliopsida; Poales; Poaceae; BOP clade; Pooideae; Triticodae; Triticeae; Triticinae; Triticum.
   CHROMOSOME: 1B
   GENE LENGTH: 3742 bp
   PROTEIN: PX domain-containing protein
   BIOLOGICAL PROCESS: phosphatidylinositol binding
   UniProt ACCESSION: A0A3B5Z651
   CULTIVAR: Artigas (PI 73046); Glenlea (CItr 17272)
   TEMPLATE: mRNA (cDNA)
   FRAGMENT LENGTH: 103 bp
   GENE REGION: 278 – 380 bp
   REFERENCE GENOME: Triticum aestivum (taxid: 4565)
   CDS sequence:
   CTTTGATAGTTCTGTTTCGCTACCTATCACTTGACTACGACTTCCGTAGAAAGAGCACA
   ACTACCACAGATCGTGATGCCAGTCGCCCACTTGCCAAAACAAA

8. **TraesCS1D02G431600.1 (*Lr46-WKRY*) - partial CDS**
   SEQUENCE ID: XM_044522263.1
   GENE SYMBOL: LOC123100317
   SOURCE: Triticum aestivum (bread wheat)
   ORGANISM: Triticum aestivum
   Eukaryota; Viridiplantae; Streptophyta; Embryophyta; Tracheophyta; Spermatophyta; Magnoliopsida; Liliopsida; Poales; Poaceae; BOP clade; Pooideae; Triticodae; Triticeae; Triticinae; Triticum.
   CHROMOSOME: 1D
   GENE LENGTH: 1226 bp
   PROTEIN: WRKY domain-containing protein
   BIOLOGICAL PROCESS: WKRY transcription factor
   UniProt ACCESSION: A0A3B6A1Y2
   CULTIVAR: Artigas (PI 73046); Glenlea (CItr 17272)
   TEMPLATE: mRNA (cDNA)
   FRAGMENT LENGTH: 119 bp
   GENE REGION: 906 – 1024 bp
   REFERENCE GENOME: Triticum aestivum (taxid: 4565)
   CDS sequence:
   TTTCTTCGCCTCTTTTGACGACGATTTTGATCATTTCTTCGAGGACGACGCCATTGGGC
   GACGGGTCTCGCTGTAGCTAGCTCTATAATATAGCTAKGCAGTACGAGAATTGGTTCCA
   C
