# Supplementary material for: Expression patterns of candidate genes for the Lr46/Yr29 “slow rust” locus in common wheat (Triticum aestivum L.) and associated miRNAs inform of the gene conferring the Puccinia triticina resistance trait
Source: PLoS One. 2024 Sep 6;19(9):e0309944. doi: 10.1371/journal.pone.0309944 (PMC11379320; doi:10.1371/journal.pone.0309944)
Supplement: S1 Table — (PDF) [file pone.0309944.s002.pdf]

Table S1. Expression of the nine *Lr46/Yr29* candidate genes in leaf tissues under *Puccinia triticina* infection.

Cultivar	Time	Gene								
		Lr46-Glu1	*Lr46-Glu2*	*Lr46-Glu3*	*Lr46-RLK1*	*Lr46-RLK2*	*Lr46-RLK3*	*Lr46-RLK4*	*Lr46-Snex*	*Lr46-WRKY*
Artigas	0 hpi	0,397	1,23	0,0561	0,05014	0,15285	0,367	0,03401	0,3761	0,05351
	6 hpi	6,703	9,859	0,2236	0,1431	N/A	2,159	0,08163	0,5118	0,13082
	12 hpi	3,825	5,759	0,2569	0,09254	0,08024	1,27	0,04744	0,4117	0,06574
	24 hpi	2,782	8,893	0,4875	0,11442	0,07759	1,69	0,10608	0,4457	0,13204
	48 hpi	2,014	3,959	0,2057	0,09472	0,04319	0,817	0,05619	0,3181	0
Artigas*	0 hpi	0,348	1,126	0,3117	0,0321	0,01264	0,495	0,27904	0,8669	0,28564
	6 hpi	0,34	0,807	0,2408	0,012	0,01135	0,337	0,18774	0,9122	0,23394
	12 hpi	0,604	1,665	0,5425	0,01364	0,02826	0,567	0,36805	1,589	0,36823
	24 hpi	0,237	0,513	0,1401	0,01917	0,00478	0,299	0,15312	0,7222	0,16446
	48 hpi	0,308	0,674	0,1849	0,01479	0,00156	0,346	0,23592	0,8982	0,08547
Glenlea	0 hpi	1,649	2,103	0,1436	0,036	0,09158	0,515	0,03196	0,3084	0,1859
	6 hpi	6,297	14,598	0,2304	0,32871	0,23868	4,465	0,09341	0,3978	0,08263
	12 hpi	1,514	6,302	0,3355	0,06937	0,08022	0,738	0,04009	0,2942	0,03148
	24 hpi	8,847	12,624	3,0216	0,08099	0,26447	3,621	0,00342	2,1263	0,25595
	48 hpi	1,526	4,313	0,1278	0,09752	0,04397	1,011	0,03725	0,3189	0
Lerma Rojo	0 hpi	0,602	0,775	0,0737	0,01565	0,05361	0,351	0,01356	0,8386	0,0456
	6 hpi	0,06	1,476	0,0247	0,06191	0,01854	0,023	0,02539	0,0691	N/A
	12 hpi	4,502	6,172	0,128	0,07642	0,06878	1,4	0,01899	0,2428	0,02315
	24 hpi	2,585	6,685	0,2885	0,07097	0,06187	0,729	0,07735	0,3118	0,14372
	48 hpi	0,134	0,784	0,0462	0,04093	0,01094	0,39	0,00572	0,1067	0
NP846	0 hpi	7,563	3,756	0,0731	0,10916	0,094	1,262	0,02476	0,5508	0,02456
	6 hpi	2,451	7,387	0,0715	0,20374	0,15891	2,225	0,02902	0,4502	0,0927
	12 hpi	4,245	6,381	0,3089	0,08094	0,03185	1,04	0,03876	0,2995	0,04291
	24 hpi	6,747	10,559	0,9552	0,25224	0,19432	3,66	0,09683	0,7786	0,12331
	48 hpi	0,359	0,813	0,1312	0,06229	0,02384	0,471	0,00945	0,1773	0
TX89D6435	0 hpi	3,382	0,687	0,0784	0,03747	0,02477	0,982	0,01192	1,1283	0,01942
	6 hpi	2,48	3,169	0,1747	0,06845	0,08006	1,637	0,05816	0,4145	0,13035
	12 hpi	5,271	3,798	0,1127	0,06134	0,05225	1,317	0,01324	0,3736	0,0177
	24 hpi	7,926	7,398	0,3013	0,17129	0,09791	3,282	0,04613	0,7777	0,02225
	48 hpi	2,673	1,363	0,15	0,05429	0,02704	1,509	0,01863	0,4177	0
