# Supplementary material for: Expression patterns of candidate genes for the Lr46/Yr29 “slow rust” locus in common wheat (Triticum aestivum L.) and associated miRNAs inform of the gene conferring the Puccinia triticina resistance trait
Source: PLoS One. 2024 Sep 6;19(9):e0309944. doi: 10.1371/journal.pone.0309944 (PMC11379320; doi:10.1371/journal.pone.0309944)
Supplement: S2 Table — The table shows the elemental contrasts for each cultivar independently in order to compare mean expression at each time point tested after inoculation to expression before inoculation. (PDF) [file pone.0309944.s003.pdf]

Supplementary Table S2. Elemental contrast values for candidate genes expression. The table shows the elemental contrasts for each cultivar independently in order to compare mean expression at each time point tested after inoculation to expression before inoculation.

Contrast	*Lr46-Glu1*	*Lr46-Glu2*	*Lr46-Glu3*	*Lr46-RLK1*	*Lr46-RLK2*	*Lr46-RLK3*	*Lr46-RLK4*	*Lr46-Snex*	*Lr46-WRKY*
Artigas 6h vs. 0h	6.31**	8.63***	0.168	0.093**	-0.0794	1.793*	0.0476	0.136	0.0773
Artigas 12h vs. 0h	3.77	3.22	0.122	0.0426	-0.0726	0.727	-0.004	0.08	-0.0287
Artigas 24h vs. 0h	2.39	7.93**	0.433	0.0568	-0.0753	1.359	0.07	0.013	0.0837
Artigas 48h vs. 0h	1.62	2.73	0.15	0.0446	-0.109*	0.451	0.0222	-0.058	-0.0535
Glenlea 6h vs. 0h	4.65*	12.49***	0.087	0.2927***	0.1471**	3.95***	0.0615	0.089	-0.1031
Glenlea 12h vs. 0h	-0.14	4.2	0.192	0.0334	-0.0114	0.224	0.0081	-0.014	-0.1538
Glenlea 24h vs. 0h	7.2***	10.52***	2.878***	0.045	0.1729**	3.107***	-0.0285	1.818***	0.07
Glenlea 48h vs. 0h	-0.12	2.21	-0.016	0.0615	-0.0476	0.496	0.0053	0.011	-0.1859*
Lerma Rojo 6h vs. 0h	-0.54	0.7	-0.049	0.0463	-0.0351	-0.328	0.0118	-0.77	0.0484
Lerma Rojo 12h vs. 0h	3.9	5.4*	0.054	0.0608	0.0152	1.049	0.0054	-0.596	-0.0224
Lerma Rojo 24h vs. 0h	1.98	5.91*	0.215	0.0553	0.0083	0.378	0.0638	-0.527	0.0981
Lerma Rojo 48h vs. 0h	-0.47	0.01	-0.028	0.0253	-0.0427	0.039	-0.0078	-0.732	-0.0456
NP846 6h vs. 0h	-5.11*	3.63	-0.002	0.0946**	0.0649	0.963	0.0043	-0.101	0.0681
NP846 12h vs. 0h	-3.32	2.62	0.236	-0.0282	-0.0621	-0.222	0.014	-0.251	0.0184
NP846 24h vs. 0h	-0.82	6.8**	0.882*	0.1431***	0.1003	2.398***	0.0721	0.228	0.0988
NP846 48h vs. 0h	-7.2***	-2.94	0.058	-0.0469	-0.0702	-0.791	-0.0153	-0.374	-0.0246
TX89D6435 6h vs. 0h	-0.9	2.48	0.096	0.031	0.0553	0.655	0.0462	-0.714	0.1109
TX89D6435 12h vs. 0h	1.89	3.11	0.034	0.0239	0.0275	0.336	0.0013	-0.755	-0.0017
TX89D6435 24h vs. 0h	4.54*	6.71**	0.223	0.1338***	0.0731	2.3**	0.0342	-0.351	0.0028
TX89D6435 48h vs. 0h	-0.71	0.68	0.072	0.0168	0.0023	0.528	0.0067	-0.711	-0.0194
Artigas* 6h vs. 0h	-0.01	-0.32	-0.071	-0.0201	-0.0013	-0.158	-0.0913	0.045	-0.0517
Artigas* 12h vs. 0h	0.26	0.54	0.231	-0.0185	0.0156	0.071	0.089	0.722	0.0826
Artigas* 24h vs. 0h	-0.11	-0.61	-0.172	-0.0129	-0.0079	-0.196	-0.1259	-0.145	-0.1212
Artigas* 48h vs. 0h	-0.04	-0.45	-0.127	-0.0173	-0.0111	-0.149	-0.0431	0.031	-0.2002*

* P<0.05; ** P<0.01; *** P<0.001
