# Supplementary material for: Expression patterns of candidate genes for the Lr46/Yr29 “slow rust” locus in common wheat (Triticum aestivum L.) and associated miRNAs inform of the gene conferring the Puccinia triticina resistance trait
Source: PLoS One. 2024 Sep 6;19(9):e0309944. doi: 10.1371/journal.pone.0309944 (PMC11379320; doi:10.1371/journal.pone.0309944)
Supplement: S3 Table — The table shows the elemental contrasts for each cultivar independently to compare the average expression at each time point tested after inoculation with the expression before inoculation. (PDF) [file pone.0309944.s004.pdf]

Supplementary Table S3. **Elemental contrast values for expression of miRNA molecules complementary to candidate genes.** The table shows the elemental contrasts for each cultivar independently to compare the average expression at each time point tested after inoculation with the expression before inoculation.

Targeted candidate genes	*Lr46-Glu2* and *Lr46-RLK2*	*Lr46-Glu2*	*Lr46-Glu2*	*Lr46-RLK3*
Contrast	tae-miR9780	tae-miR9775	tae-miR5384-3p	tae-miR164
Artigas 6h vs. 0h	-1	2	-23	-20
Artigas 12h vs. 0h	1	3	-32	187
Artigas 24h vs. 0h	1	4*	18	674***
Artigas 48h vs. 0h	6	4*	-10	217
Glenlea 6h vs. 0h	-4	1	-67	-19
Glenlea 12h vs. 0h	-3	1	-50	224
Glenlea 24h vs. 0h	-3	2	-70	6
Glenlea 48h vs. 0h	-3	3	-66	189
Lerma Rojo 6h vs. 0h	4	0	13	84
Lerma Rojo 12h vs. 0h	-3	-1	-13	176
Lerma Rojo 24h vs. 0h	6	0	9	49
Lerma Rojo 48h vs. 0h	11	0	39	283*
NP846 6h vs. 0h	-18*	6***	-36	284*
NP846 12h vs. 0h	-7	0	-12	218
NP846 24h vs. 0h	-5	-2	-45	-77
NP846 48h vs. 0h	-18*	2	-27	156
TX89D6435 6h vs. 0h	17*	-4*	-43	-381**
TX89D6435 12h vs. 0h	13	-4*	-45	-363**
TX89D6435 24h vs. 0h	18*	-1	7	-321*
TX89D6435 48h vs. 0h	17*	0	-22	-293*

* P<0.05; ** P<0.001; *** P<0.001
